# Supplementary material for: A systematic review of controlled studies of suicidal and self-harming behaviours in adolescents following bereavement by suicide
Source: PLoS One. 2021 Jul 9;16(7):e0254203. doi: 10.1371/journal.pone.0254203 (PMC8270178; doi:10.1371/journal.pone.0254203)
Supplement: S1 File — (PDF) [file pone.0254203.s001.pdf]

Suicidal and self-harming behaviours in adolescents bereaved by suicide compared to other modes of death: a systematic review

Laura del Carpio, Susan Rasmussen, Sally Paul, Abigail Paterson

Citation

Laura del Carpio, Susan Rasmussen, Sally Paul, Abigail Paterson. Suicidal and self-harming behaviours in adolescents bereaved by suicide compared to other modes of death: a systematic review. PROSPERO 2016 CRD42016051125 Available from: http://www.crd.york.ac.uk/PROSPERO/display_record.php?ID=CRD42016051125

Review question

Does bereavement by suicide lead to a greater risk of suicidal/self-harming behaviours in adolescent survivors than bereavement due to other causes?
Do the relevant studies identify factors that explain why suicide bereavement leads to suicidality/self-harm in some survivors but not others?
What types of measures are used to capture experiences of suicidal/self-harming behaviours in the literature?

Searches

The following electronic databases will be searched: MEDLINE, PsycINFO, Web of Science, and EMBASE.
Reference lists from relevant papers will also be hand searched to identify additional studies, as well as reference lists from previous reviews, and core suicidology journals.
Search terms will include variations of the kewords "bereavement", "self-harm/suicide", and "adolescent". For example, (bereave* OR grie* OR mourn*) AND (self-harm* OR self-injur* OR suicid*) AND (adolescen* OR youth*).

Studies must be published in English. There will be no restrictions on publication date.

Types of study to be included

Inclusion criteria: Included papers must be original studies published in peer-reviewed journals. They may use quantitative, qualitative, or mixed-methods approaches. Studies must compare suicide bereavement to at least one other non-suicide bereaved comparison group, and include some measurement or report of self-harm and/or suicidal behaviours. Studies must include participants between the ages of 12-18 years.
Exclusion criteria: Review articles will be excluded, and studies including non-bereaved controls. Studies with participants who do not fall within the age range of 12-18 years (i.e. solely younger children or older adults) will be excluded.

Condition or domain being studied

Bereaved adolescents.

Participants/population

Includes adolescents between the ages of 12-18 years bereaved by the death of a loved one, such as family members, partners, friends, or other close personal relationships.
Excludes studies focusing on deaths reported by the media and assisted suicides.

Intervention(s), exposure(s)

Exposure to suicide bereavement.

Comparator(s)/control

Includes: exposure to a non-suicide bereavement.
Excludes: studies of non-bereaved controls.

Context

Main outcome(s)
Self-harming behaviours or suicidal behaviours (ideation/attempts/death) following bereavement, irrespective of intent.

Timing and effect measures

Not applicable.

Additional outcome(s)
Variables that increase or decrease the likelihood of self-harming or suicidal behaviours following bereavement.

Timing and effect measures

Not applicable.

Data extraction (selection and coding)
In the first stage, the first reviewer (LdC) will complete the literature searches, remove duplicate references, and screen titles and/or abstracts of all studies to identify potential papers that meet inclusion criteria. The number of rejected articles will be recorded. In the second stage, LdC will obtain full text versions of all potential studies and assess for eligibility. Reasons for rejecting articles will be recorded. Difficult cases will be resolved by discussion with the second reviewer (SR), and unresolved cases will be referred to the third reviewer (SP).
A pre-piloted data extraction form will be completed by LdC for eligible studies. Details to be extracted include: author/s, year of publication, setting, sample characteristics (size/demographics), details of bereavement (mode of death, relationship to deceased), study design, methodology used to ascertain suicidal/self-harming behaviours, and relevant findings. The fourth reviewer (AP) will independently extract data from a proportion of the papers to check for accuracy; any discrepancies will be resolved through discussion, with second (SR) and third reviewers (SP) if needed.
Missing data will be requested from study authors.

Risk of bias (quality) assessment
Risk of bias will be assessed by the first reviewer using a pre-piloted critical appraisal tool based on Newcastle-Ottawa Quality Assessment Scales. To avoid bias, a proportion of the papers will be independently quality assessed by the fourth reviewer. Any disagreements over the risk of bias in individual papers will be resolved through discussion, and referred to the second and third reviewers if appropriate.

Strategy for data synthesis
A qualitative or narrative synthesis is planned, and will likely be structured around mode of death, relationship to the deceased, age groups, and methods used.
It is anticipated that a meta-analysis will not be feasible, given the limited research and substantial heterogeneity in research designs and methodologies across studies.

Analysis of subgroups or subsets
Subgroup analyses are planned for modes of death, relationship to the deceased, participant characteristics (age group), and methodology, should the data allow.

Contact details for further information
Laura del Carpio
laura.del-carpio@strath.ac.uk

Organisational affiliation of the review
University of Strathclyde
http://www.strath.ac.uk/

Review team members and their organisational affiliations
Miss Laura del Carpio. University of Strathclyde
Dr Susan Rasmussen. University of Strathclyde

Dr Sally Paul. University of Strathclyde
Miss Abigail Paterson. University of Strathclyde

Type and method of review

Systematic review

Anticipated or actual start date

14 November 2016

Anticipated completion date

29 February 2020

Funding sources/sponsors

This work is funded by a Wellcome Trust doctoral studentship; review to be completed as part of Ms del Carpio's PhD studies

Conflicts of interest

None known

Language

English

Country

Scotland

Stage of review

Review Ongoing

Subject index terms status

Subject indexing assigned by CRD

Subject index terms

Bereavement; Grief; Humans; Suicidal Ideation; Suicide

Date of registration in PROSPERO

22 November 2016

Date of publication of this version

09 July 2019

Revision note for this version

The protocol has been updated to reflect a focus on studies of adolescent survivors (studies that include individuals aged 12-18 years). It was decided to use the Newcastle-Ottawa Quality Assessment Scales in place of CASP checklists. Details have been added of a fourth review team member who will be assisting with data extraction and quality analyses.

Details of any existing review of the same topic by the same authors

Stage of review at time of this submission

Stage	Started	Completed
Preliminary searches	Yes	Yes
Piloting of the study selection process	Yes	No
Formal screening of search results against eligibility criteria	Yes	No
Data extraction	Yes	No
Risk of bias (quality) assessment	Yes	No
Data analysis	No	No

Revision note

The protocol has been updated to reflect a focus on studies of adolescent survivors (studies that include individuals aged 12-18 years). It was decided to use the Newcastle-Ottawa Quality Assessment Scales in place of CASP checklists. Details have been added of a fourth review team member who will be assisting with data extraction and quality analyses.

Versions

22 November 2016
09 July 2019

PROSPERO
This information has been provided by the named contact for this review. CRD has accepted this information in good faith and registered the review in PROSPERO. The registrant confirms that the information supplied for this submission is accurate and complete. CRD bears no responsibility or liability for the content of this registration record, any associated files or external websites.
