# Supplementary material for: A systematic review of controlled studies of suicidal and self-harming behaviours in adolescents following bereavement by suicide
Source: PLoS One. 2021 Jul 9;16(7):e0254203. doi: 10.1371/journal.pone.0254203 (PMC8270178; doi:10.1371/journal.pone.0254203)
Supplement: Attachment — Submitted filename: Response to Reviewers.docx [file pone.0254203.s004.docx]

**Response to Reviewers**

Reviewer #1: I have considered the revisions made by the author and although it is clear that a lot of work went into this review the fundamental design limitations have not been properly addressed.
As I noted in my previous review that heterogeneity of this kind has and can be addressed through meta-analysis, in the very field that this review sits. It is important that we continue to move the field forward, building upon existing evidence. Not doing so, adds further noise to a field that has on more than one occasion suffered from spurious evidence. This is particularly the case when the authors have sought to answer what is essentially a quantitative question (Is suicidal behaviour unique to those who are bereaved by suicide vs other causes of death) with a narrative synthesis. The methods used in this review do not align with this type of research question and I stand by my previous comment that comparison to individuals bereaved by other causes of death do not inform whether the outcomes are unique to those bereaved by suicide. This research question alone has issues since most risk factors associated with suicidal behaviour have low specificity (i.e unique to suicidal behaviour in the first place.

**Response:**

We agree that meta-analysis is a very valuable tool where appropriate. In the present manuscript, however, heterogeneous clinical characteristics (varied populations, geography), study designs (which involved different sources of bias and confounding), and exposures and outcomes measured, suggest that summary measures are not necessarily appropriate here. This has been cautioned by several authors (e.g. Borenstein, Hedges, Higgins, & Rothstein, 2009; Greco, Zangrillo, Biondi-Zoccai, & Landoni, 2013; Haidich, 2010) who highlight that the inclusion of low quality studies in a meta-analytic review, of which there are several in our manuscript, can result in biased and misleading summary measures.

Indeed, other reviews in this area also refrained from conducting pooled analyses (e.g. Andriessen, Draper, Dudley, & Mitchell, 2016; Hua, Bugeja, & Maple, 2019; Kuramoto, Brent, & Wilcox, 2009; Pitman, Osborn, King, & Erlangsen, 2014; Sveen & Walby, 2008) on the basis of variability in outcome measures, study populations, kinship relationships, and study designs, as well as concerns about the potential for confounding and selection biases to impact pooled results.

The publication by Hill et al. (2020) which offers meta-analytic findings focuses on a different study population (i.e. does not compare those exposed to suicide with those exposed to non-suicide death comparison groups), and thus faces different considerations with regards to heterogeneity. Other meta-analyses in the wider field (e.g. Witt et al., 2019) do not use similar exposure/comparison groups, and some only conduct meta-analysis on data from randomised controlled trials (e.g. Robinson et al., 2018). For these reasons, we feel justified in presenting a narrative summary of data from a strictly conducted systematic review which follows our study protocol developed a priori.

We disagree that comparison with individuals bereaved by other causes does not inform outcomes unique to those bereaved by suicide. A similar approach was taken by Sveen and Walby (2008) and Pitman et al. (2014), who also stressed that comparison with those bereaved by other causes allows for an examination of unique outcomes to suicide bereavement, rather than factors associated with the experience of bereavement in general.

We agree that bereavement by suicide would represent a risk factor with low specificity if found to be associated with self-harm or suicide-related outcomes. However, we still feel this merits further investigation, and certainly advances research in this field. It is acknowledged that research must look beyond the study of individual risk factors in isolation to predict self-harm or suicide outcomes (e.g. O'Connor & Kirtley, 2018). For this reason, we support the application of theory in research which considers the wider biopsychosocial context of individuals to better understand the complex pathways to suicide. Nevertheless, research on specific factors (such as suicide bereavement) is still beneficial to help capture this broader context of suicide.

Reviewer #2: Systematic review of adolescent suicide article REVISION
Although in my estimation this article submission has not been fully cleansed of all its minor and major imperfections, it is sufficiently on target to be of value to those attempting to make sense and coherent meaning from the large body of research out there on the effects of suicide bereavement upon adolescents, and will be of value to researchers and clinicians. The authors have worked diligently and effectively to address the criticisms offered by all reviewers and have responded satisfactorily to my own criticisms. I think there is one essential rewording required for the study abstract to make the study’s contents clearer. The first sentence of the methods section of the abstract should also include these words: “including several that extended beyond this age range.” With this amendment, I recommend acceptance of this manuscript for publication.

**Response:** We appreciate the reviewer’s comments and are pleased that they consider the manuscript worthy of acceptance for publication with their suggested amendment. In light of the changes we have now made to the manuscript following the feedback from Reviewer #3, we hope that these amendments address the reviewer’s concerns regarding the methods section of the abstract.

Reviewer #3: Dear authors thank you for your replies and for addressing my concerns. While I acknowledge that the manuscript has improved, I notice that one crucial concern has not been addressed. In my review I highlighted that several of the selected studies did not match with the age range criterium that was stipulated in the inclusion criteria. According to the inclusion criteria, selected studies “needed to include adolescent participants, who were defined as being between the ages of 12-18 years old at the time of assessment of outcomes”.
The authors replied that: “All studies chosen for inclusion needed to include at least some individuals within the stipulated 12-18 year old bracket, as per our inclusion criteria.”
I understand this reply and I agree it is a reasonable approach. However, several of the included studies explicitly excluded participants below age 18, and included participants age 18+ (as presented by the authors in Table 1). Hence, those studies must be removed from the review as they did not include or overlap with the 12-18 age range.
In their reply, authors argued that studies have used different age ranges to study groups such as ‘young people’. This may be the case. However, if the review stipulates an inclusion criterium of ages 12-18, it seems reasonable to expect that the review adheres to its inclusion criterium.

**Response:** We appreciate the reviewer’s comments on this issue. We had initially deemed that by including studies which sampled individuals aged 18 and above, this did in fact meet our inclusion criteria of 12 to 18 year olds (at least for a small number of participants in these studies). However, we have given much thought to the reviewer’s comments, and weighed our concerns regarding the applicability of the findings to adolescent populations, against the risk of loss of data regarding young people if we excluded these studies.

After much consideration, we feel that although studies which involve 18 year olds and above may represent the experience of *some* adolescents, we agree that the nine papers in question here which only sampled 18+ year olds focus primarily on the experience of older adults. For this reason, we are in agreement with the reviewer that the manuscript will be strengthened by excluding these studies, and offers more convincing evidence of the adolescent experience of bereavement following suicide or other deaths.

Following a review of the data after the removal of these nine publications, we can report that our conclusions across all outcome categories do not change materially with the exclusion of these studies. We now present an updated manuscript with amended tables, flow chart, and results sections, as well as comments added to the methods and discussion sections to reflect this change. We thank the reviewer for their feedback and feel this has improved the manuscript considerably.

**References**

Andriessen, K., Draper, B., Dudley, M., & Mitchell, P. B. (2016). Pre- and postloss features of adolescent suicide bereavement: A systematic review. *Death Studies, 40*(4), 229-246. doi:10.1080/07481187.2015.1128497

Borenstein, M., Hedges, L. V., Higgins, J. P. T., & Rothstein, H. R. (2009). When Does it Make Sense to Perform a Meta-Analysis? *Introduction to Meta-Analysis*: John Wiley & Sons, Ltd.

Greco, T., Zangrillo, A., Biondi-Zoccai, G., & Landoni, G. (2013). Meta-analysis: pitfalls and hints. *Heart, Lung and Vessels, 5*(4), 219-225.

Haidich, A. B. (2010). Meta-analysis in medical research. *Hippokratia, 14*(Suppl 1), 29-37.

Hill, N. T. M., Robinson, J., Pirkis, J., Andriessen, K., Krysinska, K., Payne, A., . . . Lampit, A. (2020). Association of suicidal behavior with exposure to suicide and suicide attempt: A systematic review and multilevel meta-analysis. *PLoS Med, 17*(3), e1003074. doi:10.1371/journal.pmed.1003074

Hua, P., Bugeja, L., & Maple, M. (2019). A systematic review on the relationship between childhood exposure to external cause parental death, including suicide, on subsequent suicidal behaviour. *J Affect Disord, 257*, 723-734. doi:10.1016/j.jad.2019.07.082

Kuramoto, S. J., Brent, D. A., & Wilcox, H. C. (2009). The impact of parental suicide on child and adolescent offspring. *Suicide and Life-Threatening Behavior, 39*(2), 137-151.

O'Connor, R. C., & Kirtley, O. J. (2018). The integrated motivational-volitional model of suicidal behaviour. *Philosophical Transactions of the Royal Society B, 373*(1754), 1-10. doi:10.1098/rstb.2017.0268

Pitman, A., Osborn, D., King, M., & Erlangsen, A. (2014). Effects of suicide bereavement on mental health and suicide risk. *The Lancet Psychiatry, 1*(1), 86-94. doi:10.1016/s2215-0366(14)70224-x

Robinson, J., Bailey, E., Witt, K., Stefanac, N., Milner, A., Currier, D., . . . Hetrick, S. (2018). What Works in Youth Suicide Prevention? A Systematic Review and Meta-Analysis. *EClinicalMedicine, 4-5*, 52-91. doi:10.1016/j.eclinm.2018.10.004

Sveen, C.-A., & Walby, F. A. (2008). Suicide survivors' mental health and grief reactions: A systematic review of controlled studies. *Suicide and Life-Threatening Behavior, 38*(1), 13-29.

Witt, K., Milner, A., Spittal, M. J., Hetrick, S., Robinson, J., Pirkis, J., & Carter, G. (2019). Population attributable risk of factors associated with the repetition of self-harm behaviour in young people presenting to clinical services: a systematic review and meta-analysis. *European Child & Adolescent Psychiatry, 28*(1), 5-18. doi:10.1007/s00787-018-1111-6
